# Predicting the efficacy of glucocorticoids in pediatric primary immune thrombocytopenia using plasma proteomics

**DOI:** 10.3389/fimmu.2023.1301227

**Published:** 2023-12-14

**Authors:** Qingqing Cao, Haiyan Zhu, Wei Xu, Rongrong Zhang, Yun Wang, Zhaofang Tian, Yufang Yuan

**Affiliations:** ^1^ Department of Pediatrics, The Affiliated Huaian No.1 People’s Hospital of Nanjing Medical University, Huai’an, China; ^2^ Department of Neonatology, The Affiliated Huaian No.1 People’s Hospital of Nanjing Medical University, Huai’an, China

**Keywords:** immune thrombocytopenia, children, glucocorticoids, 4D-DIA, plasma biomarker

## Abstract

**Objective:**

Primary immune thrombocytopenia (ITP) is the most common acquired autoimmune bleeding disorder among children. While glucocorticoids are the primary first-line treatment for ITP treatment, they prove ineffective in certain patients. The challenge of identifying biomarkers capable of early prediction regarding the response to glucocorticoid therapy in ITP persists. This study aimed to identify ideal biomarkers for predicting glucocorticoid efficacy in patients with ITP using plasma proteomics.

**Methods:**

A four-dimensional data-independent acquisition approach was performed to determine the differentially expressed proteins in plasma samples collected from glucocorticoid-sensitive (GCS) (n=18) and glucocorticoid-resistant (GCR) (n=17) children with ITP treated with prednisone. The significantly differentially expressed proteins were selected for enzyme-linked immunosorbent assay validation in a cohort conprising 65 samples(30 healthy controls, 18 GCS and 17 GCR children with ITP). Receiver operating characteristics curves, calibration curves, and clinical decision curve analysis were used to determine the diagnostic efficacy of this method.

**Results:**

47 differentially expressed proteins (36 up-regulated and 11 down-regulated) were identified in the GCR group compared with the GCS group. The significantly differentially expressed proteins myosin heavy chain 9 (MYH9) and fetuin B (FETUB) were selected for enzyme-linked immunosorbent assay validation. The validation results were consistent with the proteomics analyses. Compared with the GCS group, the GCR group exhibited a significantly reduced the plasma concentration of MYH9 and elevated the plasma concentration of FETUB. Furthermore, the receiver operating characteristics curves, calibration curves, and clinical decision curve analysis demonstrated good diagnostic efficacy of these validated biomarkers.

**Conclusion:**

This study contributes to the establishment of objective biological indicators for precision therapy in children with ITP. More importantly, the proteins MYH9 and FETUB hold potential as a foundation for making informed decisions regarding alternative treatments for drugresistant patients, thereby preventing treatment delays.

## 1 Introduction

Primary immune thrombocytopenia (ITP) is the most common autoimmune bleeding disorder among children, characterised by excessive platelet destruction and impaired platelet production, resulting in platelet counts falling below 100×10^9^/L (1, 2). Clinical manifestations include petechiae, purpura, mucosal bleeding, and, in rare cases, intracranial haemorrhage, often accompanied by a health-related decline in the patient’s quality of life (3). The incidence of ITP in children is approximately 1.9-6.4 per 100,000 individuals (4, 5), surpassing that observed in adults. Glucocorticoids constitute a cornerstone in the first-line treatment of ITP, being the most cost-effective option (6). They function by inhibiting autoantibody production by lymphocytes and curtail macrophage activity, thereby reducing platelet destruction (7). However, approximately 20% of patients are resistant to glucocorticoids and experience recurrent bleeding symptoms, thereby affecting their quality of life (8). Moreover, continuous glucocorticoids therapy does not confer benefits to glucocorticoid-resistant (GCR) patients, and while delaying the need for treatment, patients experience various side effects such as hypertension, hyperglycaemia, and osteoporosis (9–12). Therefore, biomarkers are needed to identify GCR patients with ITP prior to commencing long-term medication to reduce the incidence of serious adverse events, improve the quality of life of the affected patients, and encourage the early use of alternative therapies before the onset of bleeding symptoms.

In recent years, there has been a continuous influx of reports examining biomarkers for assessing glucocorticoid efficacy in ITP treatment. Unfortunately, a consensus in clinical practice has not yet emerged. Furthermore, most of these investigations have centred on adults, leaving uncertainties regarding their applicability to children. Using real-time polymerase chain reaction assays, it was observed that stromal cell-derived factor 1 and miRNA-125a-5p were closely associated with glucocorticoid sensitivity, while adenosine triphosphate-binding cassette subfamily B member 1 was associated with glucocorticoid resistance (12–14). Naguib et al. and Li et al., through enzyme-linked immunosorbent assay (ELISA) analysis, reported increased levels of nuclear factor-κB (NF-κB) and intercellular adhesion molecule-1 in the GCR patients with ITP (13, 15). Notably, a flow cytometry analysis of patients with ITP demonstrated a lowered interleukin (IL)-10 to IL-17 ratio in the GCR group (16). Additionally, one study using time-of-flight mass spectrometry (MS) analysis revealed that glucocorticoid-sensitive (GCS) individuals exhibited significantly higher levels of sirtuin 1 and hypoxia-inducible-factor 1A, suggesting a potential link to their favourable response to glucocorticoid therapy (17, 18).

Proteomics technology, particularly the data-independent acquisition (DIA) technology, has been relatively underutilised in the study of markers associated with glucocorticoid efficacy in children with ITP. The four-dimensional (4D)-DIA proteomics technology uses the parallel accumulation-serial fragmentation (PASEF) combined with DIA (diaPASEF) acquisition mode of the timsTOF Pro2 series mass spectrometer to conduct differential quantitative proteomic analysis. The diaPASEF acquisition mode is a combination of the DIA acquisition mode and the PASEF technology, offering the advantage of acquiring DIA data without compromising window cycling speed. This concurrently reduces spectral complexity and enhances ion utilisation, resulting in a comprehensive enhancement of proteomics in terms of coverage depth, sensitivity, and throughput (19, 20). This study aimed to employ 4D-DIA quantitative proteomics technology to analyse the differentially expressed protein profiles in GCS and GCR children. Subsequently, these findings were validated using ELISA. This endeavour contributes valuable insights for identifying biomarkers associated with the efficacy of glucocorticoids in children with ITP.

## 2 Patients and methods

### 2.1 Study cohort and grouping

This study encompassed a cohort of 35 children with a primary diagnosis of ITP who were hospitalised at the Department of Paediatrics within Huaian No.1 People’s Hospital of Nanjing Medical University and 30 age-and sex-matched healthy volunteers at the same time as the outpatient clinic from 1 February to 1 July 2023. All participants met the diagnostic criteria for ITP as outlined in the ITP Neunert C practice guidelines (6). The inclusion criteria were as follows: children who were recently diagnosed with ITP; those who had not undergone glucocorticoid and/or intravenous gammaglobulin treatment; and those capable of adhering to a regular follow-up schedule. The exclusion criteria were as follows: congenital or secondary thrombocytopaenia; congenital immunodeficiency disorders; and the inability to maintain regular follow-up appointments or instances of missed visits.

Prednisone treatment was administered according to guideline recommendations (21), with a specific dose of 2 mg/kg/day (with a maximum of 60 mg/day) for 2 weeks. Based on the response to glucocorticoid therapy, the patients were divided into two groups, namely the GCS group and the GCR group. The criteria for group assignment were as follows: 1) The GCS group: Platelet counts ≥30×10^9^/L and at least two times greater than the baseline level, with no observable bleeding manifestations after completing the 2-week prednisolone treatment (administered at 2 mg/kg, followed by tapering); 2) The GCR group: Platelet counts below<30×10^9^/L, less than two times the baseline, or displaying bleeding symptoms after the 2-week prednisone treatment. Informed consent was obtained from all study participants, who also provided their signatures on a written informed consent form. This study adhered to the guidelines of the Declaration of Helsinki and was approved by the ethics committee of the Affiliated Huaian No.1 People’s Hospital of Nanjing Medical University.

### 2.2 Samples collection

Peripheral blood of approximately 3 mL were obtained from patients with ITP before initiating treatment. These samples were collected into dipotassium ethylene diamine tetraacetic acid (EDTA) premixed vacuum tubes and subsequently subjected to centrifugation at 3000 r/min for 10 min at room temperature. After centrifugation, the supernatant was carefully collected to isolate plasma specimens, which were then frozen in a -80°C refrigerator for future use. Following a randomised selection process, three plasma specimens were selected from each group for 4D-DIA analysis (Wuhan Maiwei Metabolism Co., Ltd., Wuhan, China).

### 2.3 Main reagents

Albumin from bovine serum was procured from Wuhan Chucheng Zhengmao Science and Technology Engineering Co., Ltd. DL-Dithiothreitol were procured from Solarbio, while EDTA, Xylene brilliant cyaninG-250, Sodium dodecyl sulfate, Thiourea, and Acetone were procured from Sinopharm. Iodoacetamide was procured from Aladdin, and Phenylmethanesulfonyl fluoride was procured from Xiya Reagent. Tetraethylammonium bromide and Urea were procured from Sigma. Trypsin was procured from Promega, and the protein marker was procured from Fementas. Lastly, the bicinchoninic acid (BCA) protein quantification kit was procured from Biyuntian. ELISA kits for myosin heavy chain 9 (MYH9) and fetuin B (FETUB) were procured from Wuhan Fearn and Eliot Biotechnology Co.

### 2.4 DIA

#### 2.4.1 Sample preparation

Frozen peripheral plasma specimens were lysed at room temperature, followed by the removal of high-abundance proteins using the ProteoMiner™ Protein Enrichment Small Volume Kit (Bio-Rad). The resulting eluate was collected to determine the total protein concentration through BCA protein quantification analysis. An aliquot of protein solution was taken based on its concentration, and the volume was adjusted to 200 µL with 8 M urea. Subsequently, it was reduced with 10 mM dithiothreitol for 45 min at 37°C and alkylated with 50 mM iodoacetamide for 15 min under dark conditions at room temperature. For precipitation, pre-cooled acetone, added in four-fold volume to the protein solution, was employed. This precipitation process was performed at -20°C for 2 h. Following centrifugation, the protein precipitates were collected and resuspended in a 200 µL solution comprising 25 mM amine bicarbonate solution and 3 µL of trypsin. The mixture was allowed to undergo digestion at 37°C overnight. After digestion, peptides from each sample were subjected to desalination using a C18 column, concentrated via vacuum centrifugation, and subsequently redissolved in a 0.1% (v/v) formic acid solution.

#### 2.4.2 Liquid chromatography-MS/MS detection

The samples were separated using a nanolitre flow rate NanoElute ultra-high-performance liquid chromatography system. Mobile phase A comprised a 0.1% formic acid aqueous solution, while mobile phase B comprised a 0.1% formic acid ethene solution (acetonitrile 100%). An autosampler loaded the samples onto an analytical column (25 cm × 75 µm, C18 packing 1.6 µm) for separation. The analytical column was maintained at 50°C, and the sample volume was set at 200 ng, with a flow rate of 300 nL/min over a 60-min gradient. The liquid-phase gradient program was as follows: 0 min–45 min, linear increase of liquid B from 2% to 22%; 45 min–50 min, linear gradient from 22% to 35% for liquid B; 50 min–55 min, linear gradient from 35% to 80% for liquid B; 55 min–60 min, liquid B was maintained at 80%. After chromatographic separation, the mixed samples were subjected to MS data collection in data-dependent acquisition (dda) PASEF mode using the timsTOF Pro2 mass spectrometer. The analysis featured a 60-min effective gradient, positive ion detection mode, a parent ion scanning range of 100–1700 m/z, ion mobility range (1/*K_0_*) of 0.7–1.4 Vs/*cm* ^2^, ion accumulation and release time of 100 ms, and nearly 100% ion utilisation. Parameters included a capillary voltage of 1500 V, a drying gas rate of 3 L/min, and a drying temperature of 180°C. In the ddaPASEF acquisition mode, parameters included 10 MS/MS scans with a total cycle time of 1.17 s, charge range of 0–5, dynamic exclusion time of 0.4 min, ion target intensity set at 10,000, ion intensity threshold at 2500, collision-induced dissociation fragmentation energy of 42 eV, and an isolation window setting of 2 for <700 Th and 3 for >700 Th. For the diaPASEF acquisition mode, parameters encompassed a mass range of approximately 400–1200, mobility range of 0.7–1.4 Vs/*cm* ^2^, mass width of 25Da, a mass overlap of 0.1, 32 mass steps per cycle, and two mobility windows, resulting in a total of 64 acquisition windows. The average acquisition period was 1.8 s.

#### 2.4.3 Database search and quantification

The library search software employed in the study was DIA-NN (v1.8.1). For library searching, the Libraryfree method was used with specific parameters. The database used was swissprot_Homo_sapiens_9606_20376.fasta database (20376 entries). A deep learning-based parameter was activated to predict a spectral library. The match-between-runs option was selected to create a spectral library using DIA data and reanalyse the DIA data to obtain protein quantification. Precursor ions and protein-level false discovery rates were filtered at 1%.

#### 2.4.4 Bioinformatic analysis

Comprehensive functional annotation of identified differential proteins included Gene Ontology (GO) classification (http://geneontology.org/) and the Kyoto Encyclopaedia of Genes and Genomes (KEGG) pathway (https://www.genome.jp/kegg/). Protein-protein interaction (PPI) analysis were applied to find the interactions among all DEPs by using the STRING database (https://string-db.org/).

### 2.5 ELISA

The procured ELISA kits were validated against MYH9 and FETUB, respectively, according to the manufacturer’s instructions. Absorbance values of standards and samples were read using a microplate reader at a wavelength of 450 nm.

### 2.6 Statistical analysis

Data were statistically analysed using GraphPad Prism 8.0(San Diego, CA, USA) and SPSS 26.0 (Chicago, IL, USA). Measurement data were tested for normality using the Shapiro–Wilk method, and normally distributed data are expressed as the mean ± standard deviation (SD). Comparisons between groups were made using the independent samples t-test. Data that did not conform to normal distribution are expressed as medians (quartiles) using, and between-group comparisons were made using the Mann–Whitney U test. Count data are expressed as the number of instances (percentage), and between-group comparisons were made using Fisher’s exact test and Chi-square test. Generated subject work characteristics (receiver operating characteristics [ROC]) curves, calibration curves, and clinical decision curve analysis (DCA) were also plotted to assess the model’s efficacy. *P*-values of <0.05 were considered statistically significant.

## 3 Results

### 3.1 Patient characteristics

This study included 30 healthy controls, 18 GCS and 17 GCR children with ITP. **Table 1** presents demographic details and clinical characteristics in children with ITP. Notably, there were no statistically significant differences between GCS and GCR groups in terms of sex, age and body weight. There was no statistically significant difference in gender and age between ITP patients and healthy controls (see **Supplementary Materials**).

**Table 1:** Patient characteristics.

Variables	GS group (n=18)	GR group (n=17)	t/F	*P*
Gender, n(%)			-	>0.999
Male	8 (44.44%)	8 (43.75%)		
Female	10 (55.56%)	9 (56.25%)		
Age, years (mean ± SD)	4.972 ± 3.216	6.353 ± 2.978	1.316	0.197
Weight, kg (mean ± SD)	21.60 ± 10.00	28.74 ± 17.67	1.481	0.148
Platelet counts, x10^9^/L (mean ± SD)	10 ± 7.874	16.71 ± 8.528	2.419	0.021

### 3.2 4D-DIA analysis results

**Figure 1:**
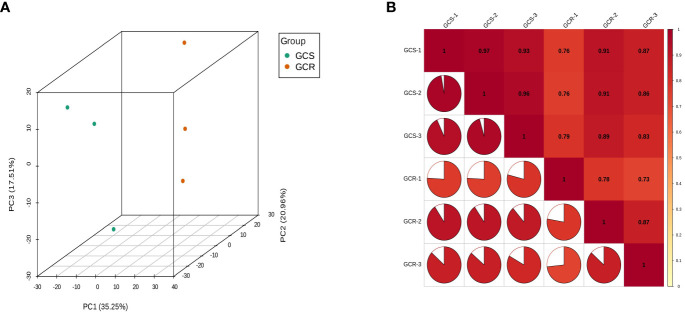
Quality assessment of quantitative results. **(A)** Principal component analysis: where PC1 represents the first principal component, PC2 represents the second principal component, and PC3 represents the third principal component; **(B)** Correlation analysis: the horizontal and vertical coordinates represent the names of the samples, and the change in colour from red to yellow represents the change in correlation from high to low. The size of the fan area in the figure represents the size of the correlation coefficient of the corresponding horizontal and vertical coordinate samples; the number in the figure represents the correlation coefficient of the corresponding horizontal and vertical coordinate samples.

Principal component analysis revealed sample dispersion between the GCS and GCR groups, with strong clustering observed within each group (**Figure 1A**). These findings underscore significant differences between the two groups. Furthermore, when evaluating the Pearson correlation of protein abundances among all sample pairs using a heat map, it was evident that the Pearson correlation coefficient for protein abundance exceeded 0.7 (**Figure 1B**). This high correlation suggests a significant consistency in protein expression across all samples.

#### 3.2.1 GO classification and the KEGG pathway analysis of differentially expressed proteins

**Figure 2:**
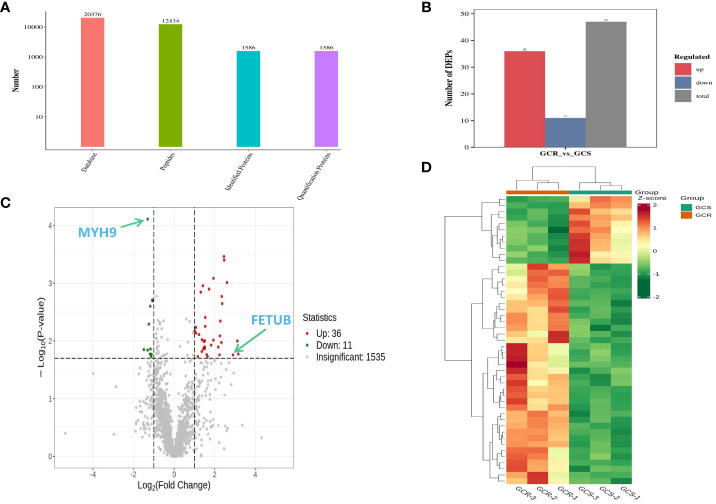
Plasma differential protein test results of sensitive and resistant groups of children with ITP. **(A)** Statistical results of mass spectrometry analysis data; **(B)** Results of differential proteins after screening conditions with protein expression FC>2.0 or <0.5 and P-value<0.02; **(C)** Volcano plot of the plasma differential proteins in the sensitive and resistance groups, where the horizontal coordinate represents the log_2_(FC), the vertical coordinate represents -log_10_(P*-*value), and the red and green scatters represent up- and down-regulation of the differential proteins, respectively; **(D)** Clustering heat map, where red indicates up-regulation and green indicates down-regulation, and the shades of the colours indicate varying degrees of up- and down-regulation.

Three plasma samples selected from each group were analysed using the NanoLC-MS/MS protein assay technology to compare GCS and GCR children. A total of 1586 quantifiable proteins were detected (**Figure 2A**). Using screening criteria of protein expression fold change (FC) >2.0 or <0.5 with a significance level of *P*<0.02, a set of 47 differential proteins was identified (**Figure 2B**). Among these proteins, 36 proteins were up-regulated and 11 proteins were down-regulated compared with the GCS group. For a visual representation, the volcano plots illustrate the significant up-regulation of the protein FETUB and the down-regulation of the protein MYH9 (**Figure 2C**). The heatmap of the differential proteins demonstrated that the expression levels of the differential proteins differed significantly between the GCS and GCR groups (**Figure 2D**).

**Figure 3:**
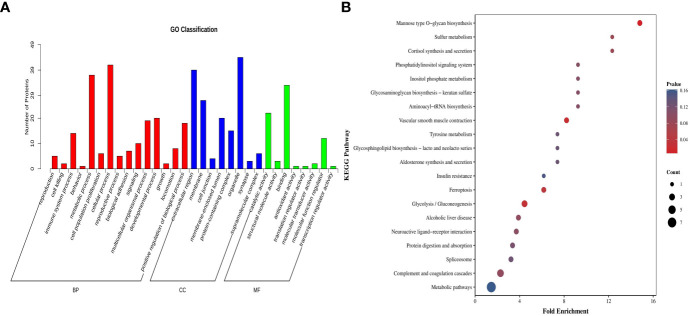
Bioinformatics analysis of plasma differential protein detection results in the sensitive and resistant groups of children with ITP. **(A)** GO classification bar graph, where the horizontal coordinates represent secondary GO entries, the vertical coordinates represent the number of differentially expressed proteins in a particular GO entry, and the different colours of the bar represent up- and down-regulation. **(B)** KEGG enrichment analysis graph, where the horizontal coordinates represent the enrichment folds and the vertical coordinates represent the KEGG pathway; the bubble colour indicates the enrichment degree, and the bubble size indicates the number of proteins enriched to this entry.

The GO enrichment analysis comprises three parts, namely biological process, cellular components, and molecular functions. Regarding biological processes, the differentially expressed proteins were primarily associated with cellular protein metabolism, negative regulation of carbohydrate derivative metabolism, and the regulation of protein hydrolysis, among others. Concerning cellular components, these proteins predominantly reside in various cellular locales, such as cellular membranes, organelles, extracellular regions, and protein-containing complexes. In terms of molecular functions, the differential proteins were primarily characterised by enzyme inhibitor activity, peptidase regulatory activity, and endopeptidase regulatory activity (**Figure 3A**). The KEGG database was used to analyse the pathway enrichment of the differential proteins. The results of this analysis revealed that the differential proteins were primarily enriched in glycolysis/gluconeogenesis and cellular tight junction pathways (**Figure 3B**).

#### 3.2.2 Protein-protein interaction analysis

**Figure 4:**
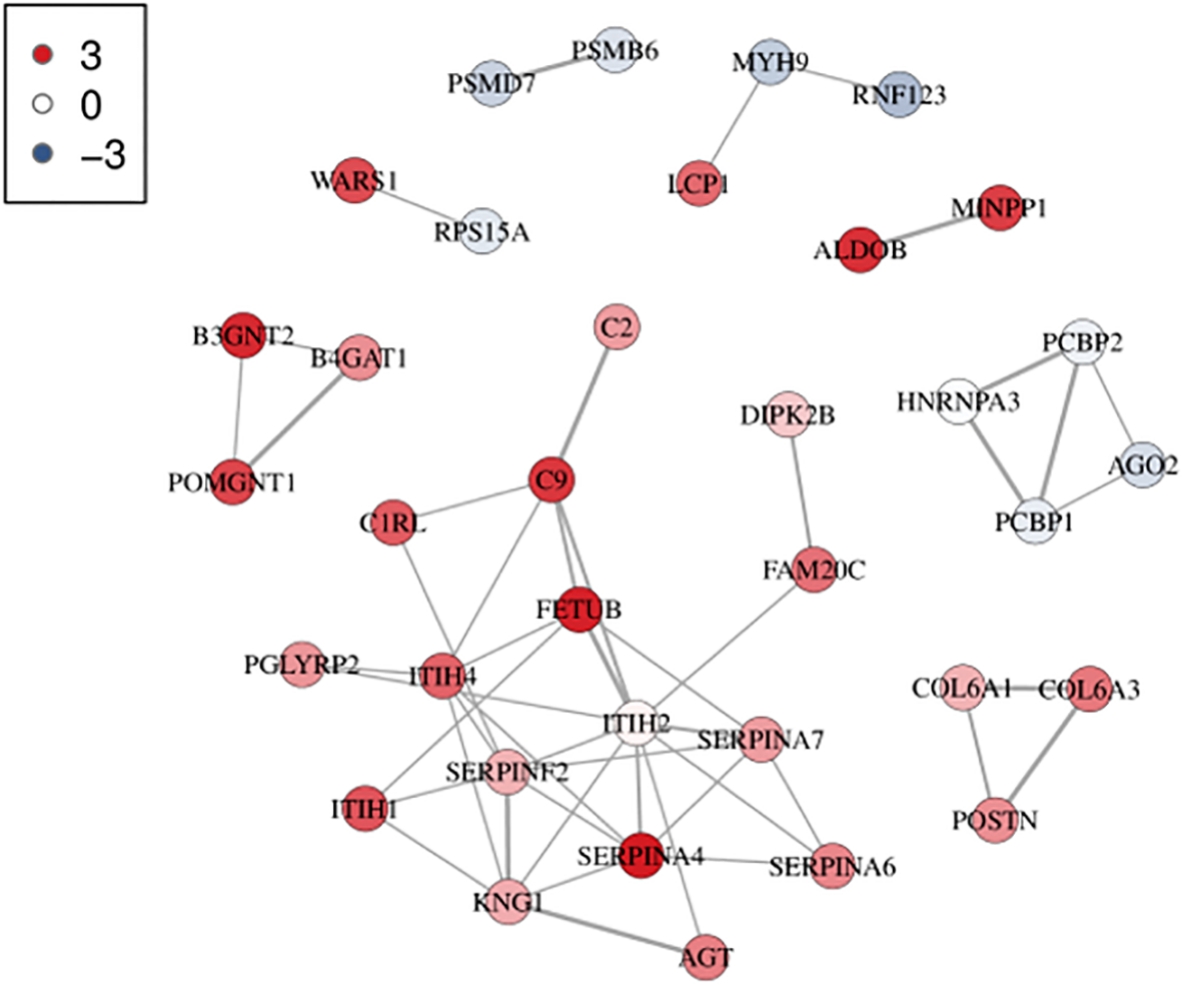
Protein interaction network analysis diagram. Each node represents a protein, and the lines between the nodes represent interaction relationships. The more the lines, the stronger the interaction relationship. The thicker the line, the more credible the interaction. The color represents the level of differential protein expression. Red indicates upregulation of differential proteins, and blue indicates downregulation of differential proteins. The darker the color, the denser the relationship. The network consists of 35 nodes and 49 edges with an average node degree of 2.8.

Based on the GO and KEGG analyses, differential protein interactions network analysis was performed by applying the interactions in the STRING protein interactions database (http://string-dborg). Subsequently, an interaction network diagram was constructed using Cytoscape. The results of PPI analysis demonstrated that the screened differentially expressed proteins form a complex regulatory network containing 35 nodes and 49 edges with the average degree of 2.8. The PPI analysis revealed that MYH9 and FETUB exhibited a higher degree of connectivity with other proteins within the network, suggesting that MYH9 and FETUB might be key proteins influencing the efficacy of glucocorticoids in ITP (**Figure 4**).

### 3.3 ELISA results

**Figure 5:**
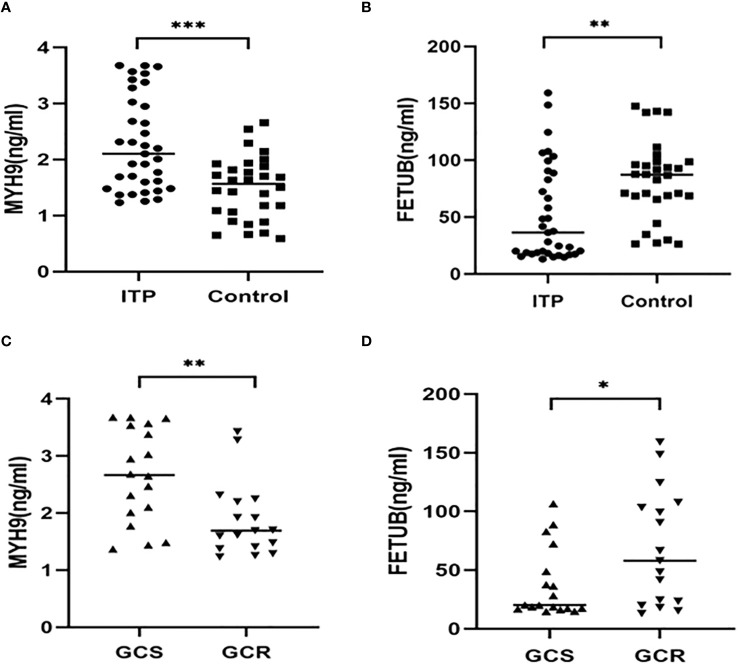
The plasma levels of MYH9 and FETUB in children with ITP (n = 35) and healthy volunteers (n = 30). **(A, B)** Comparison of MYH9 and FETUB plasma levels in children with ITP versus the healthy controls. **(C, D)** Comparison of MYH9 and FETUB plasma levels in the GCS group(n = 18) and the GCR group(n = 17). *P<0.05,**P<0.01,***P<0.001.

The upregulated protein FETUB and downregulated protein MYH9 were selected for verification in validation cohorts (n= 65, 30 healthy controls, 18 GCS and 17 GCR children with ITP) using ELISA. Compared with the healthy control group, the levels of MYH9 were significantly increased (*P*<0.001) and FETUB were significantly decreased (*P*<0.01) in patients with ITP (**Figures 5A, B**). Compared with the GCS group, the GCR group exhibited a significantly reduced the plasma concentration of MYH9 and elevated the plasma concentration of FETUB (**Figures 5C, D**).

### 3.4 Efficacy of the model

**Figure 6:**
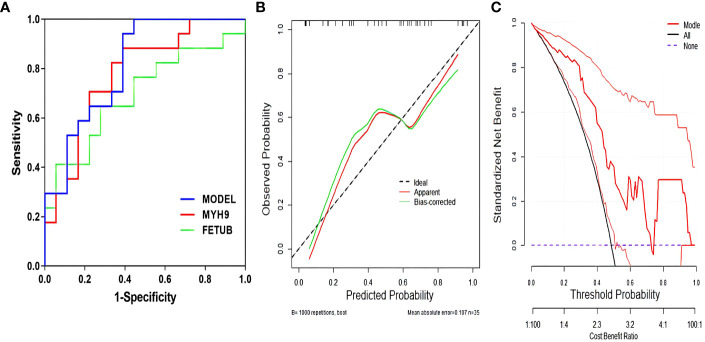
Efficacy of the model of predicting glucocorticoid resistance. **(A)** ROC curves for predicting glucocorticoid resistance by each metric; **(B)** Calibration curves for predicting glucocorticoid resistance; **(C)** DCA for predicting glucocorticoid resistance.

There were multifactorial logistic regression analysis concerning factors influencing glucocorticoid resistance (**Table 2**). Notably, FETUB and MYH9 emerged as statistically significant factors in the model (*P*<0.05). The findings suggest that as the level of MYH9 decreases and the level of FETUB increases, the risk of glucocorticoid resistance in patients increases. It displays the ROC curves for predicting glucocorticoid resistance using FETUB and MYH9 (**Figure 6A**). The areas under the curve for FETUB, MYH9, and their combined prediction of glucocorticoid resistance were 0.696, 0.778, and 0.814, respectively. These values yielded corresponding *P*-values < 0.05, underscoring the statistical significance of each indicator in predicting glucocorticoid resistance (see **Supplementary Materials**). For FETUB, a cut-off value of 39.649 was established, resulting in a sensitivity of 64.7% and a specificity of 72.2%. Meanwhile, MYH9 had a cut-off value of 2.392, with a sensitivity of 88.2% and a specificity of 61.1%. When both indicators were jointly considered, the cut-off value was 0.272, yielding a sensitivity of 100.0% and a specificity of 55.6% for predicting glucocorticoid resistance.

**Table 2:** Multifactorial logistic regression analysis affecting glucocorticoid resistance.

Variables	B	SE	Waldχ^2^	*P*	OR (95% CI)
FETUB	0.025	0.012	4.333	0.037	1.026 (1.001-1.051)
MYH9	-1.631	0.654	6.225	0.013	0.196 (0.054-0.705)
Constant	2.263	1.337	2.863	0.091	-

It illustrates the calibration curve and DCA of the model for predicting glucocorticoid resistance (**Figures 6B, C**). The Hosmer-Lemeshow goodness-of-fit test for the logistic regression model yielded χ2 value of 13.731, with a corresponding *P*-values of 0.056, which exceeded 0.05, indicating the efficacy of the model. Furthermore, the clinical DCA of the predictive model demonstrated that the model’s performance was superior within the probability range of 0.05 to 0.75.

## 4 Discussion

ITP is the most common bleeding disorder among children. The heterogeneity within ITP renders the response anticipation of treatment responses a challenging endeavour. In instances where children exhibit resistance to hormone-based therapies for ITP, there is an increased risk of enduring prolonged and ultimately chronic disease. Therefore, there is an urgent need for biomarkers that can predict glucocorticoid responsiveness, offering objective biological indicators for the precise management of children with ITP. Moreover, these biomarkers might serve as a basis for alternative therapeutic approaches for the GCR patients, thereby avoiding delays in treatment initiation.

There are few studies examining plasma proteomics concerning the efficacy of glucocorticoids in children with ITP. In this study, plasma specimens from patients with ITP in the GCS and GCR groups were analysed proteomically. This led to the initial identification of a set of biomarkers closely associated with cytoskeleton formation, protein hydrolysis, and enzyme activities. These findings were derived from the results obtained through GO analysis, KEGG signalling pathway analysis, and PPI analysis. Among these identified biomarkers, 36 differential proteins were up-regulated in expression in the GCR group, with FETUB demonstrating a particularly significant difference (FC=7.471). Meanwhile, 11 proteins were also found to be down-regulated in expression, with MYH9 having the smallest *P*-values (*P*<0.00008).

MYH9, a 230 kDa cytoskeletal protein, participates in several vital cellular processes such as cell adhesion, migration, and signalling (22–24). Numerous studies have reported that MYH9 affects the haematopoietic system, leading to impaired bone marrow haematopoiesis (25–28). Human MYH9 gene mutations are associated with a group of autosomal dominant disorders, collectively known as MYH9-related disorders, which are characterised by thrombocytopenia (29). Experimental models with point mutations in the MYH9 gene have exhibited reduced platelet adhesion and intracellular interactions (30). An investigation by An et al. indicated that MYH9 knockout mice experienced severe haematopoietic impairment, resulting in diminished whole blood cell counts and bone marrow dysfunction. Additionally, MYH9 gene deletion was observed to affect the repopulation ability of haematopoietic stem/progenitor cells while increasing apoptosis, implying its involvement in organismal haematopoiesis (31). Furthermore, proteomic analysis revealed the significant potential of MYH9 in early prediction of GCR childhood nephrotic syndrome, positioning it as a potential candidate biomarker for evaluating glucocorticoid efficacy (32–34). Our proteomics study also found that the difference in MYH9 protein levels between the GCS and GCR groups was statistically significant. MYH9 levels in ITP patients were significantly higher than those in the healthy controls. More importantly, the close relationship between MYH9 levels and response to steroid therapy has been demonstrated in ITP. ROC curve analysis revealed a cut-off value of 2.392 and an ROC value of 0.778, with a high sensitivity of 88.2% and a specificity of 61.1%. These findings collectively propose MYH9 as a potential biomarker for predicting the response to steroid therapy in ITP.

Human fetoglobulin encompasses fetuin A (FETUA) and FETUB (35). Predominantly originating from the liver, FETUA is also found in limited quantities within monocytes/macrophages (36, 37). Existing literature underscores FETUA as a multifunctional plasma protein, pivotal in neutrophil and platelet degranulation (38). Moreover, FETUA serves as an endogenous ligand for toll-like receptor (TLR) 4 (TLR4), actively participating in the inflammatory response. By binding to the extracellular structural domain of TLR4, it activates NF-κB signalling, subsequently inducing the release of pro-inflammatory cytokines from macrophages. The involvement of FETUA in TLR activation and consequent inflammatory signalling can contribute to glucocorticoid resistance (39, 40). Although fewer studies have been conducted on FETUB, its structural homology to FETUA suggests a potentially similar role. Research has indicated the value of FETUB in predicting glucocorticoid resistance in paediatric nephrotic syndrome (41). In the present study, DIA analysis revealed significant differences in the levels of FETUB protein between the GCS and GCR groups. Notably, FETUB exhibited higher levels in the GCR group compared with the GCS group, with this difference attaining statistical significance. Moreover, PPI analysis indicated that FETUB exhibited more extensive associations with other proteins, suggesting its potential role as a key player in this context. ELISA analysis found that the FETUB levels in ITP patients were significantly lower than those in the healthy controls. In ITP patients, the levels of FETUB in the GCR group is significantly higher than the GCS group, which is consistent with the results of DIA analysis, underscoring the potential significance of FETUB in the mechanism of glucocorticoid action in ITP. It is worth noting that most existing studies on FETUB and glucocorticoid resistance have mainly focused on urologic diseases. Further exploration is warranted to understand the precise mechanisms through which FETUB contributes to the development of glucocorticoid resistance in the context of ITP.

The ROC curve analysis in this study revealed that the area under the curve value for the combined prediction of glucocorticoid sensitivity using MYH9 and FETUB was significantly superior to that of MYH9 or FETUB alone, suggesting that the combination of these metrics has a higher predictive value for glucocorticoid resistance. Furthermore, results from the calibration curves and DCA indicated that the combined prediction model incorporating MYH9 and FETUB exhibited commendable diagnostic efficacy. In summary, by analysing the plasma proteomic profiles of different therapeutic responses to glucocorticoids in children with ITP, two important proteins, MYH9 and FETUB, were screened in this study, confirming their role as potential biomarkers of glucocorticoid efficacy in children with ITP. These findings provide novel insights and important information for further study of glucocorticoid resistance in ITP. However, this study has some limitations. First, it is a single-centre study with a small sample size. Second, our study only revealed the relationship between MYH9 and FETUB and glucocorticoid sensitivity, necessitating further experimental exploration to uncover the underlying mechanisms.

## Data availability statement

The original contributions presented in the study are publicly available. This data can be found here: ProteomeXchange through the PRIDE database with the accession number PXD046260.

## Ethics statement

The studies involving humans were approved by the ethics committee of the Affiliated Huaian No.1 People’s Hospital of Nanjing Medical University. The studies were conducted in accordance with the local legislation and institutional requirements. Written informed consent for participation in this study was provided by the participants’ legal guardians/next of kin.

## Author contributions

QC: Data curation, Validation, Writing – original draft. HZ: Conceptualization, Methodology, Software, Writing – review & editing. WX: Investigation, Methodology, Writing – review & editing. RZ: Investigation, Methodology, Writing – review & editing. YW: Investigation, Methodology, Writing – review & editing. ZT: Conceptualization, Funding acquisition, Methodology, Supervision, Writing – review & editing. YY: Conceptualization, Funding acquisition, Methodology, Supervision, Writing – review & editing.
