# Supplementary material for: Predicting the efficacy of glucocorticoids in pediatric primary immune thrombocytopenia using plasma proteomics
Source: Front Immunol. 2023 Dec 14;14:1301227. doi: 10.3389/fimmu.2023.1301227 (PMC10757608; doi:10.3389/fimmu.2023.1301227)
Supplement: Supplementary file 1 [file Table_1.docx]

Supplementary Materials

**47 differential proteins**

| Gene | FC | *P*-value | Regulated |
| --- | --- | --- | --- |
| SERPINA4 | 8.976531425 | 0.016930787 | up |
| PRDX2 | 8.674201157 | 0.010051454 | up |
| **FETUB** | 7.47147334 | 0.017506982 | up |
| B3GNT2 | 6.087268835 | 0.000978526 | up |
| ALDOB | 5.536090124 | 0.00039857 | up |
| TGFBR3 | 5.491085382 | 0.00034532 | up |
| C9 | 5.175041296 | 0.002264821 | up |
| MINPP1 | 5.093190421 | 0.001699468 | up |
| POMGNT1 | 5.033777051 | 0.010688782 | up |
| WARS1 | 4.855887291 | 0.004533737 | up |
| TAFAZZIN | 4.790981937 | 0.008190978 | up |
| ITIH1 | 4.721441597 | 0.017440719 | up |
| C1RL | 4.516776325 | 0.01275751 | up |
| ITIH4 | 3.83998234 | 0.000827105 | up |
| LCP1 | 3.823454046 | 0.009706755 | up |
| FAM20C | 3.542955026 | 0.01183824 | up |
| COL6A3 | 3.30454749 | 0.001266501 | up |
| AGT | 3.132726064 | 0.01865077 | up |
| SERPINA6 | 3.038654211 | 0.01729292 | up |
| B4GAT1 | 2.899160103 | 0.00393273 | up |
| POSTN | 2.898332454 | 0.005608007 | up |
| PGLYRP2 | 2.858242836 | 0.012715459 | up |
| SERPINA7 | 2.847892947 | 0.009945907 | up |
| C2 | 2.841518035 | 0.013592975 | up |
| GNPTG | 2.775306548 | 0.010252048 | up |
| MTAP | 2.759899707 | 0.01307646 | up |
| KNG1 | 2.72713651 | 0.013818427 | up |
| IGLV3-27 | 2.674171003 | 0.001111689 | up |
| COL6A1 | 2.591405083 | 0.009534848 | up |
| SERPINF2 | 2.5766125 | 0.015307175 | up |
| VASN | 2.488601632 | 0.001431537 | up |
| DIPK2B | 2.343553715 | 0.007761576 | up |
| KRTDAP | 2.252524802 | 0.018656009 | up |
| BPNT2 | 2.108548884 | 0.007322724 | up |
| ITIH2 | 2.105521513 | 0.005851971 | up |
| KCTD12 | 2.028662684 | 0.006785109 | up |
| HNRNPA3 | 0.478524895 | 0.002030011 | down |
| ADH1B | 0.475338623 | 0.001963734 | down |
| PCBP2 | 0.461558835 | 0.017072327 | down |
| PCBP1 | 0.456604902 | 0.018742643 | down |
| RPS15A | 0.445859036 | 0.013850193 | down |
| PSMB6 | 0.442434148 | 0.002512009 | down |
| AGO2 | 0.438995451 | 0.0168819 | down |
| PSMD7 | 0.422163028 | 0.005132746 | down |
| **MYH9** | 0.405091668 | 7.79E-005 | down |
| OXSR1 | 0.404425696 | 0.014468346 | down |
| RNF123 | 0.355255672 | 0.014162802 | down |

**Comparing Patients with ITP and Healthy Controls: Demographics and Clinical Features**

| **Variables** | **ITP（n=35）** | **Controls(n=30）** | **t/***χ^2^* | ***P*** |
| --- | --- | --- | --- | --- |
| Gender, n(%) |  |  | 0.006 | 0.939 |
| Male | 16（45.71％） | 14（53.33％） |  |  |
| Female | 19（54.29％） | 16（46.67％） |  |  |
| Age, years(mean±SD) | 5.643±3.136 | 4.467±1.889 | 1.793 | 0.078 |
| Platelet counts, x10^9^/L(mean±SD) | 13.26±8.763 | 259.6±81.1 | 17.88 | ＜0.0001 |

**ELISA results**

| group | FETUB(ng/ml) | MYH9(ng/ml) |
| --- | --- | --- |
| GCS | 18.712 | 1.368 |
| GCS | 28.179 | 2.949 |
| GCS | 16.288 | 2.309 |
| GCS | 37.528 | 1.772 |
| GCS | 88.699 | 2.103 |
| GCS | 36.354 | 2.009 |
| GCS | 18.712 | 3.675 |
| GCS | 48.946 | 1.438 |
| GCS | 20.240 | 3.377 |
| GCS | 72.396 | 3.024 |
| GCS | 14.891 | 3.535 |
| GCS | 106.560 | 2.680 |
| GCS | 14.715 | 3.568 |
| GCS | 20.134 | 1.481 |
| GCS | 16.958 | 3.677 |
| GCS | 82.998 | 3.659 |
| GCS | 17.538 | 2.469 |
| GCS | 17.343 | 2.649 |
| GCR | 99.442 | 1.480 |
| GCR | 103.440 | 1.232 |
| GCR | 48.438 | 2.248 |
| GCR | 66.611 | 1.919 |
| GCR | 15.462 | 1.375 |
| GCR | 19.943 | 1.693 |
| GCR | 124.566 | 1.614 |
| GCR | 13.085 | 1.254 |
| GCR | 159.179 | 3.424 |
| GCR | 23.653 | 1.289 |
| GCR | 90.541 | 3.277 |
| GCR | 107.749 | 1.408 |
| GCR | 24.507 | 2.314 |
| GCR | 148.553 | 1.598 |
| GCR | 41.769 | 2.199 |
| GCR | 58.020 | 1.702 |
| GCR | 18.129 | 1.923 |
| Control | 29.924 | 1.422 |
| Control | 68.736 | 1.935 |
| Control | 91.711 | 0.843 |
| Control | 82.801 | 2.542 |
| Control | 92.761 | 0.649 |
| Control | 82.801 | 1.634 |
| Control | 92.761 | 1.684 |
| Control | 34.828 | 1.065 |
| Control | 93.534 | 1.445 |
| Control | 87.779 | 1.923 |
| Control | 68.978 | 1.087 |
| Control | 104.992 | 1.771 |
| Control | 70.99 | 0.687 |
| Control | 44.416 | 1.816 |
| Control | 86.826 | 1.178 |
| Control | 111.506 | 0.884 |
| Control | 27.265 | 2.292 |
| Control | 87.574 | 1.702 |
| Control | 26.206 | 2.659 |
| Control | 68.555 | 0.66 |
| Control | 95.09 | 1.874 |
| Control | 142.194 | 1.179 |
| Control | 98.529 | 0.588 |
| Control | 142.108 | 1.501 |
| Control | 143.064 | 1.63 |
| Control | 96.015 | 1.717 |
| Control | 71.051 | 0.899 |
| Control | 147.626 | 2.14 |
| Control | 26.468 | 1.392 |
| Control | 65.63 | 2.006 |

**The ROC curve analysis of MYH9 and FETUB for prediction of GCR.**

| **Biomarkers** | ***95% CI*** | ***P*** | **Threshold value** | **Sensitivity(％）** | **Specificity(％)** |
| --- | --- | --- | --- | --- | --- |
| FETUB | 0.517-0.875 | 0.048 | 39.649 | 64.7 | 72.2 |
| MYH9 | 0.622-0.933 | 0.005 | 2.392 | 88.2 | 61.1 |
| MODEL | 0.673-0.955 | 0.002 | 0.272 | 100.0 | 55.6 |

**The Youden index by FETUB and MYH9 combined**

| The coordinates of the curve |  |  |  |  |  |
| --- | --- | --- | --- | --- | --- |
| variable | a is positive when it is greater than or equal to this value | Sensitivity | 1 - Specificity | Specificity | The Youden index |
|  | 0.2715613 | 1 | 0.444 | 0.556 | 0.556 |
|  | 0.3090176 | 0.941 | 0.389 | 0.611 | 0.552 |
|  | 0.2313865 | 1 | 0.5 | 0.5 | 0.5 |
|  | 0.2976098 | 0.941 | 0.444 | 0.556 | 0.497 |
|  | 0.3562032 | 0.882 | 0.389 | 0.611 | 0.493 |
|  | 0.1898372 | 1 | 0.556 | 0.444 | 0.444 |
|  | 0.4165156 | 0.824 | 0.389 | 0.611 | 0.435 |
|  | 0.5953057 | 0.647 | 0.222 | 0.778 | 0.425 |
|  | 0.6291341 | 0.588 | 0.167 | 0.833 | 0.421 |
|  | 0.65973 | 0.529 | 0.111 | 0.889 | 0.418 |
|  | 0.16711 | 1 | 0.611 | 0.389 | 0.389 |
|  | 0.4459075 | 0.765 | 0.389 | 0.611 | 0.376 |
|  | 0.4898065 | 0.706 | 0.333 | 0.667 | 0.373 |
|  | 0.5848511 | 0.647 | 0.278 | 0.722 | 0.369 |
|  | 0.6129776 | 0.588 | 0.222 | 0.778 | 0.366 |
|  | 0.6399629 | 0.529 | 0.167 | 0.833 | 0.362 |
|  | 0.6776868 | 0.471 | 0.111 | 0.889 | 0.36 |
|  | 0.1518536 | 1 | 0.667 | 0.333 | 0.333 |
|  | 0.4672591 | 0.706 | 0.389 | 0.611 | 0.317 |
|  | 0.5414942 | 0.647 | 0.333 | 0.667 | 0.314 |
|  | 0.6886251 | 0.412 | 0.111 | 0.889 | 0.301 |
|  | 0.838343 | 0.294 | 0 | 1 | 0.294 |
|  | 0.0996413 | 1 | 0.722 | 0.278 | 0.278 |
|  | 0.7095069 | 0.353 | 0.111 | 0.889 | 0.242 |
|  | 0.7547836 | 0.294 | 0.056 | 0.944 | 0.238 |
|  | 0.9262071 | 0.235 | 0 | 1 | 0.235 |
|  | 0.051633 | 1 | 0.778 | 0.222 | 0.222 |
|  | 0.7357015 | 0.294 | 0.111 | 0.889 | 0.183 |
|  | 0.9399412 | 0.176 | 0 | 1 | 0.176 |
|  | 0.0409536 | 1 | 0.833 | 0.167 | 0.167 |
|  | 0.9447659 | 0.118 | 0 | 1 | 0.118 |
|  | 0.0384613 | 1 | 0.889 | 0.111 | 0.111 |
|  | 0.9578664 | 0.059 | 0 | 1 | 0.059 |
|  | 0.0362772 | 1 | 0.944 | 0.056 | 0.056 |
